# Epidemiology of malaria in Rohingya refugee camps in Bangladesh within 2017–2020

**DOI:** 10.1186/s12936-023-04688-y

**Published:** 2023-09-28

**Authors:** Md. Ariful Anwar Khan, Richard James Maude, Sharmin Musa, Hamida Khanum

**Affiliations:** 1 Department of Zoology, Government Hazi Muhammad Mohsin College, Chattogram, Bangladesh; 2 Mahidol Oxford Tropical Medicine Research Unit, Faculty of Tropical Medicine, Mahidol University, Bangkok, Thailand; 3 Centre for Tropical Medicine and Global Health, Nuffield Department of Medicine, University of Oxford, Oxford, UK; 4 The Open University, Milton Keynes, UK; 5 Department of Zoology, University of Dhaka, Dhaka, Bangladesh

**Keywords:** Epidemiology, Malaria, Refugees

## Abstract

**Background:**

Malaria causes significant morbidity and mortality in tropical and sub-tropical regions, particularly in humanitarian emergencies including refugee camps in malaria endemic areas. An epidemiological investigation was conducted on malaria disease distribution and risk factors in the world’s largest refugee settlement, the Rohingya refugee camps on the south-eastern border area of Bangladesh, within 2017–2020.

**Methods:**

From February 2017 to March 2020, 30,460 febrile patients were tested for malaria using light microscopy and rapid diagnostic tests. Most were self-presenting symptomatic patients and a minority were from door-to-door malaria screening. Diagnostic tests were done by trained medical technologists upon the advice of the concerned physicians in the camps. Test positivity rate (%) and annual parasite incidence were calculated and compared using chi-squared (χ ^2^) test or odds ratios.

**Results:**

The overall average annual test positivity rate (TPR) was 0.05%. TPR was highest in people who had travelled to the forest in the previous 2 months, at 13.60%. Cases were clustered among male adults aged 15–60 years. There were no cases among children under five years or pregnant women and no deaths from malaria.

**Conclusion:**

This study found very few malaria cases among Rohingya refugees with the majority of cases being imported from hilly forested areas, which were thus assumed to act as the reservoir for transmission.

## Background

Malaria remains a major cause of morbidity and mortality in tropical and sub-tropical regions and the most deadly mosquito-borne infectious disease. In 2020, globally there were an estimated 241 million malaria cases and 627,000 deaths with the majority in Africa followed by Asia [1]. Human movement is a major contributor to changes in transmission within and between countries through circulation between endemic areas, reintroduction to formally endemic regions and introduction to new areas [2]. Surveillance for malaria in mobile and migrant populations is more challenging than in static populations and their contribution to disease burden may be under-recognized. Since 2017, more than 1.3 million Rohingya refugees migrated from Myanmar to Bangladesh [3]. Many of these came from malaria endemic regions of Myanmar, thus there was concern that they could bring with them a substantial burden of malaria infections. The area they migrated into in Bangladesh was also endemic for malaria, with potential for local transmission among this incoming population. A previous study in north-east Bangladesh described similar importation from neighbouring India [4]. Thus an epidemiological survey was conducted to quantify the burden of malaria among the Rohingya refugee population in south-east Bangladesh to understand the risk factors for infection.

## Methods

**Fig. 1:**
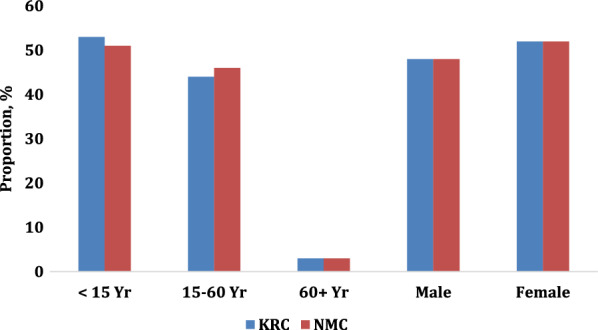
Demographic breakdown of the population in each camp. (Adapted from [8])

From March 2017 to February 2020, unselected individuals self-presenting to Primary Health Care Centers (PHCC) in Kutupalong registered camp (KRC, population 18,223), Ukhiya upazila and Nayapara mega camp (NMC, population 68,274), Teknaf upazila, both in Cox’s Bazar district, were tested for malaria using rapid diagnostic test (RDT) or light microscopy of peripheral blood as part of routine healthcare. Of the 34 refugee camps and/or makeshift settlements in Ukhiya and Teknaf, these two camps were the largest at the time of the study, and their demographic breakdown is shown in Fig. 1.

Criteria for malaria testing were tympanic temperature > 37.5° C and any age or sex [5, 6]. This passive case detection comprised 96% of tested individuals. An additional 4% of tested individuals were identified during door to door visits by the health workers using the same criteria. The tests were all done in the concerned PHCC in the camp and the data were recorded and stored in malaria surveillance registers on paper before being transferred to a secure electronic database. Two types of RDT were used, as provided by the National Malaria Elimination Programme: SD Bio Line Malaria Pf/Pv, a one-step malaria Anti-Pf/Pv (HRP-2/pLDH) test kit (Alere Medical Pvt. Ltd, Haryana, India) during 2017–2019, and Biocredit Malaria Ag Pf/Pan (HRP-2/pLDH) test kit (RapiGene, INC, Gyeonggi, Republic of Korea) during 2019–2020. Anonymized and routinely collected data on age, sex, pregnancy, travel to forests in the previous 2 months, and use of bed nets were also analysed. Test positivity rate (TPR), proportion (%), and annual parasite incidence (API) were calculated. Chi-squared (*χ*^2^) test and odds ratio (OR) were used to compare groups and assess potential risk factors. Data were also analyzed by year, month, and season as pre monsoon (hot, March-June), monsoon (wet, July-October), and post monsoon (cold and dry, November-February) [7].

## Results

**Fig. 2:**
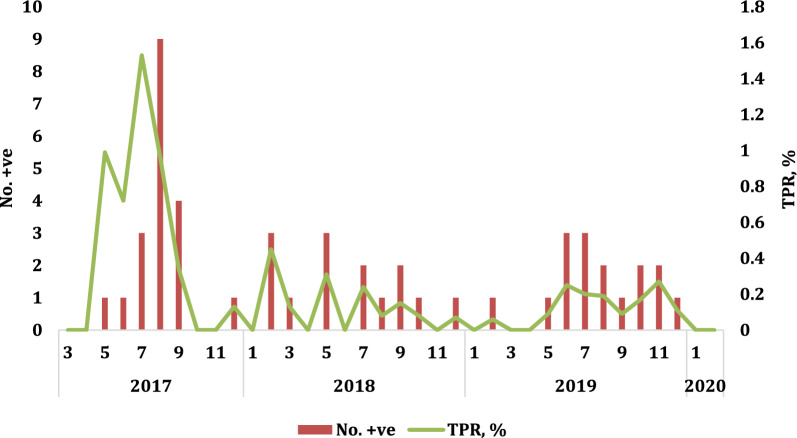
Overall number of confirmed cases and TPR by month from 2017 to 2020

Overall, 30,460 people were tested for malaria, of which 1940 were in KRC and 28,520 in NMC. Testing results are summarized in Table 1 and TPR by month in Fig. 2. Of these, 49 (0.16%) had a positive test and there were no deaths from malaria. *Plasmodium falciparum* mono infection was found in 53% (TPR 0.09%), 37% *Plasmodium vivax* (TPR 0.06%), and mixed *P. falciparum / P. vivax* in 10% (TPR 0.02%). The API was 0.19 per 1000 population.

**Table 1:** Summary of annual malaria testing results from 2017 to 2020

Groups	Indicator	2017–2018					2018–2019					2019–2020				
		No. Test	% of total	No. +ve	TPR, %	%	No. Test	% of total	No. +ve	TPR %	%	No. Test	% of total	No. +ve	TPR, %	%
Age	< 1 year	48	1	0	0	0	58	0.5	0	0	0	65	0.5	0	0	0
	1–4 year	366	5	0	0	0	356	3	0	0	0	457	4	0	0	0
	5–14 yr	1867	28	1	0.05	5	2888	25	2	0.07	17	2303	19	0	0.00	0
	15–60 yr	4387	65	21	0.48	95	8419	72	10	0.12	83	9091	76	15	0.16	100
	60 + yr	36	0.5	0	0.00	0	50	0.4	0	0.00	0	69	0.6	0	0.00	0
Gender	M.	2997	45	19	0.63	86	5710	49	8	0.14	67	5120	43	8	0.16	53
	F.	3707	55	3	0.08	14	6061	51	4	0.07	33	6865	57	7	0.10	47
Risk	Preg.	287	14	0	0	0	643	16	0	0	0	525	13	0	0	0
	Bed net use	321	10	0	0	0	668	17	0	0	0	869	17	0	0	0
	FL	32	0.5	11	34.38	58	42	0.01	4	9.52	33	51	1	2	3.92	13
Diagnostic	Micro.	2675	40	2	0.07	9	4599	39	0	0.00	0	5258	44	0	0.00	0
	RDT	4029	60	20	0.50	91	7172	61	12	0.17	100	6727	56	15	0.22	100
Species	Pv	6704	100	6	0.09	27	11,771	100	6	0.05	50	11,985	100	6	0.05	40
	Pf	6704	100	15	0.22	68	11,771	100	3	0.03	25	11,985	100	8	0.07	53
	Mixed	6704	100	1	0.01	1	11,771	100	3	0.03	25	11,985	100	1	0.01	7

TRP was higher by RDT (0.25%) compared to microscopy (0.04%, *p* < 0.001).

**Fig. 3:**
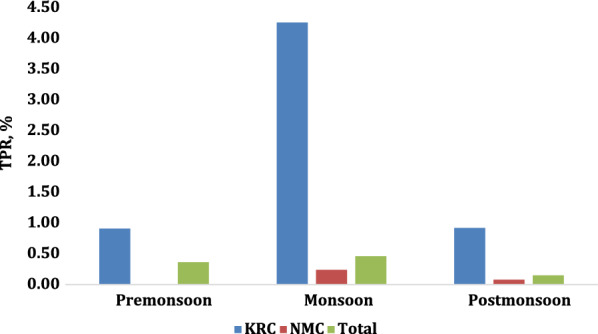
Seasonal malaria TPR, 2017–2018

**Fig. 4:**
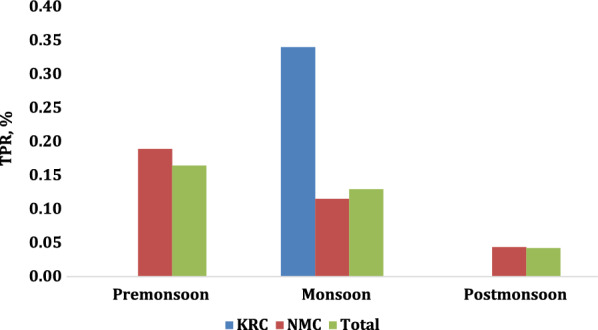
Seasonal malaria TPR, 2018–2019

**Fig. 5:**
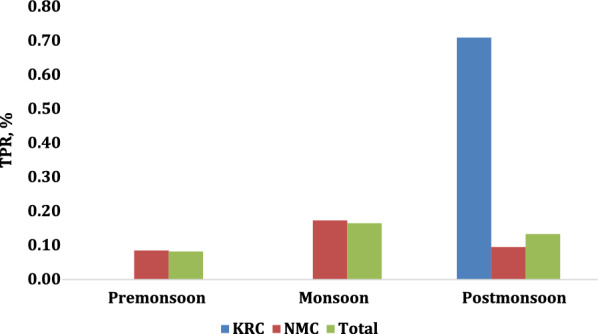
Seasonal malaria TPR, 2019–2020

There was no consistent seasonal pattern of TPR in 2017–2018, 2018–2019, or 2019–2020 in either camp or combined (Figs. 3, 4 and 5). Overall, TPR was 0.13% in pre-monsoon, 0.23% in monsoon, and 0.09% in post-monsoon seasons (*p* = 0.03). Overall annual malaria TPR was highest in 2017–2018 at 0.33% (*p* < 0.001). From 2017 to 2020, overall TPR was higher in KRC than in NMC, *p* = 0.01.

**Fig. 6:**
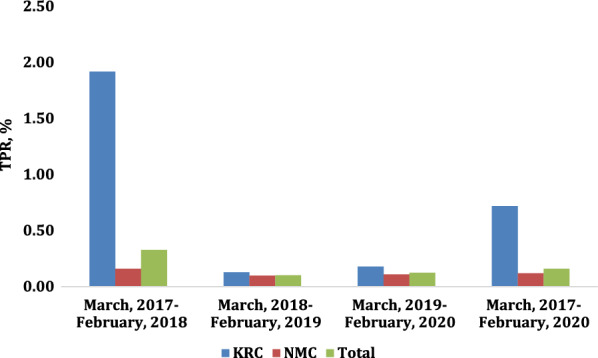
Annual and overall malaria TPR from 2017 to 2020

Malaria TPR was higher in KRC than in NMC during 2017–2018 (1.92% vs. 0.16%, *p* < 0.001) but not in the other years (0.13% vs. 0.10%, *p* = 0.79, in 2018–2019; 0.18% vs. 0.12%, *p* = 0.71, in 2019–2020; Fig. 6).

TPR was highest among people aged 15–60 years (OR (95% CI) = 6 (2–19), *p* = 0.01), and males (OR (95% CI) = 3 (2–6), *p* < 0.001).

**Fig. 7:**
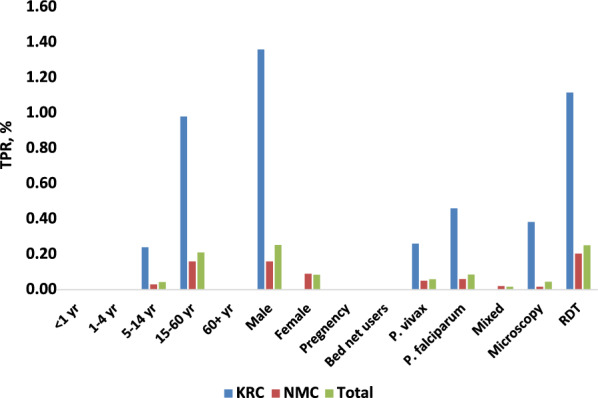
Overall malaria TPR by demographics, parasite species, and diagnostic test method from 2017 to 2020

TPR among people who had travelled to the forest in the previous two months (13.60%) was much higher than in those who had not (0.11%, OR (95% CI) = 120 (60–238), *p* < 0.001). TPR among the 1858 people who slept under bed nets was 0%, all cases occurring in people who did not use bed nets, (*p* = 0.01, Fig. 7). All malaria positive cases were treated (with chloroquine plus primaquine for *P. vivax* and artemether-lumefantrine for *P. falciparum*). No cases presented again with malaria, thus all were presumed cured.

## Discussion

This study found very few malaria cases and no deaths among 86,490 Rohingya refugees in 2 camps in Southeast Bangladesh. Furthermore, it demonstrated that the majority of these cases were likely locally infected after arriving in Bangladesh thus importation of malaria from Myanmar in this group is low. This is in contrast to other trans-border elimination settings in Africa and South-east Asia where incidence among refugees was relatively high [8, 9]. The high proportion of cases that had travelled to the forest in Bangladesh warrants further investigation as, similar to other neighbouring countries, this is where most malaria transmission occurs [10, 11]. In particular, more detail about where they went and why, would help to inform strategies to further reduce transmission. It would also be informative to have more information about previous malaria treatment of participants in Myanmar and/or Bangladesh before refugees arrived at the camps as this would have reduced importation. These findings are in agreement with other studies in Rohingya refugees during this period. In 2018, a household survey among refugee settlements in the same area found no malaria positive people among 1239 (all aged 1–14 years) tested [7]. IgG seroprevalence in the same study was higher among those who had arrived during the high transmission season in Myanmar, interpreted by the authors as suggesting increased exposure to infection during their transit. In another study of Forcefully Displaced Myanmar Nationals (FDMN) presenting to public health facilities in Cox’s Bazar, Bangladesh, of 9,421 individuals seeking healthcare during July 2018-December 2019, only 3 had malaria [7].

There were no positive cases among children under five years or pregnant women, both groups with higher risk of severe malaria. This is likely because these groups had not travelled to the forest and thus been exposed to transmission. That were no cases who presented twice suggests high cure rates by the national standard treatment regimens of chloroquine plus primaquine and artemether-lumefantrine. This suggests that anti-malarial resistance to these drugs is not a significant problem in this area, as has been shown in other studies [12–16].

This study had a number of limitations. The data used were routinely collected from passive surveillance and house-to-house screening. Thus, very limited information was available about each case. In particular more details about when individuals travelled from Myanmar, where and when they had gone to the forest in Bangladesh, and previous malaria history would have been very informative. It was also only possible to include data from 2 out of 34 camps with 6.7% of the estimated 1.3 million total refugees. The unselected nature of participants, however, means that the sample should be representative of the included populations.

The findings imply that preventing travel to the forest, provision of transmission prevention measures to people visiting forests, and testing and treating those who have visited forests, should be prioritized to minimize malaria in this population. Further study is needed to identify where transmission is happening and the best prevention methods for this group.

## Conclusion

Malaria among Rohingya refugees in 2 camps in Southeast Bangladesh is rare and mostly acquired by adult males during visits to the forest inside Bangladesh. Thus importation of malaria from Myanmar by this group is likely to be low and efforts to reduce malaria in this population should be targeted at adult forest goers.

## Data Availability

Not applicable.

## Abbreviations

AAI: Average annual incidence
API: Annual parasite incidence
FL: Forest linked
FDMN: Forcibly displaced Myanmar nationals
HRP-2: Histidine rich protein-2
KRC: Kutupalong registered camp
NMC: Nayapara mega camp
NMEP: National Malaria Elimination Programme
PHCC: Primary health care centre
pLDH: Plasmodial lactate dehydrogenase
TPR: Test positivity rate
UNHCR: United Nations High Commission for Refugees
WHO: World Health Organization
